# Crystal structures and Hirshfeld surface analyses of hypoxanthine salts involving 5-sulfosalicylate and perchlorate anions

**DOI:** 10.1107/S2056989022004753

**Published:** 2022-05-13

**Authors:** Udhayasuriyan Sathya, Jeyaraman Selvaraj Nirmalram, Sundaramoorthy Gomathi, Franc Perdih, Samson Jegan Jennifer, Ibrahim Abdul Razak

**Affiliations:** a Centre for Research and Development, PRIST Deemed to be University, Thanjavur 613 403, Tamil Nadu, India; b Department of Chemistry, Periyar Maniammai Institute of Science and Technology, Thanjavur 613 403, Tamil Nadu, India; c Faculty of Chemistry and Chemical Technology, University of Ljubljana, Vecna, pot 113, PO Box 537, SI-1000 Ljubljana, Slovenia; d X-ray Crystallography Unit, School of Physics, University Sains Malaysia, 11800, USM, Penang, Malaysia; Vienna University of Technology, Austria

**Keywords:** crystal structure, hydrogen bonding, Hirshfeld surface analysis, hypoxanthine

## Abstract

Two salts of 1,9-di­hydro­purin-6-one (hypoxanthine), namely, 6-oxo-1,9-di­hydro­purin-7-ium 5-sulfosalicylate dihydrate, C_5_H_5_N_4_O^+^·C_7_H_5_O_6_S^−^·2H_2_O, (**I**), and 6-oxo-1,9-di­hydro­purin-7-ium perchlorate monohydrate, C_5_H_5_N_4_O^+^·ClO_4_^−^·H_2_O, (**II**), have been synthesized and characterized using single-crystal X-ray diffraction and Hirshfeld analysis. In both salts, the hypoxanthine mol­ecule is protonated at the N7 position of the purine ring. In salt (**I**), the cation and anion are connected through N—H⋯O inter­actions. The protonated hypoxanthine cations of salt (**I**) form base pairs with another symmetry-related hypoxanthine cation through N—H⋯O hydrogen bonds with an *R*_2_^2^(8) ring motif, while in salt (**II**), the hypoxanthine cations are paired through a water mol­ecule *via* N—H⋯O and O—H⋯N hydrogen bonds with an *R*_3_^3^(11) ring motif. The packings within the crystal structures are stabilized by π–π stacking inter­actions in salt (**I**) and C—O⋯π inter­actions in salt (**II**). The combination of several inter­actions leads to the formation of supra­molecular sheets extending parallel to (010) in salts (**I**) and (**II**). Hirshfeld surface analysis and fingerprint plots reveal that O⋯H/H⋯O contacts play the major role in the crystal packing of each of the salts, with a 54.1% contribution in salt (**I**) and 62.3% in salt (**II**).

## Abstract 2

The main inter­molecular inter­actions in the two title salts are O⋯H/H⋯O contacts, as revealed by Hirshfeld surface analyses.

## 1. Chemical context

1,9-Di­hydro­purin-6-one (hypoxanthine, C_5_H_4_N_4_O), a notable purine-based nucleotide (Emel’yanenko *et al.*, 2017 ▸), is present in the anti­codon as nucleoside inosine in t-RNA (Costas & Acevedo-Chávez, 1997 ▸; Holley *et al.*, 1965 ▸; Stryer, 1988 ▸; Plekan *et al.*, 2012 ▸; Hughes, 1981 ▸; Schmalle *et al.*, 1988 ▸). Hypoxanthine and xanthine are significant as drugs in the treatment of infections like gout and xanthinuria. Hypoxanthine is additionally utilized against hypoxia and is known to repress the impact of few medications (Dubler *et al.*, 1987*a* ▸,*b* ▸; Biradha *et al.*, 2010 ▸).

Hypoxanthine (HX), a potential oxygen-free radical generator, is a strong agent against cancer cells (Susithra *et al.*, 2018 ▸; Latosińska *et al.*, 2014 ▸; Rutledge *et al.*, 2007 ▸). The presence of the imine group in its structure is responsible for its pharmacological activity. Hypoxanthine can exist in two stable tautomers, *viz.* as the oxo-N7(H) form and as the oxo-N9(H) form. When hypoxanthine inter­acts with strong acids, it becomes protonated at position N7 or N9. A limited number of hypoxanthine salts like hypoxanthine nitrate (Cabaj & Dominiak, 2021 ▸; Cabaj *et al.*, 2019 ▸) and hypoxanthine hydro­chloride monohydrate (Sletten & Jensen, 1969 ▸) have been reported so far in the literature.

The current article reports the crystal structures of hypoxanthinium 5-sulfosalicylate dihydrate, (**I**), and hypoxan­thin­ium perchlorate monohydrate, (**II**), salts and the noncovalent inter­actions that govern their crystal packings.

## 2. Structural commentary

**Figure 1:**
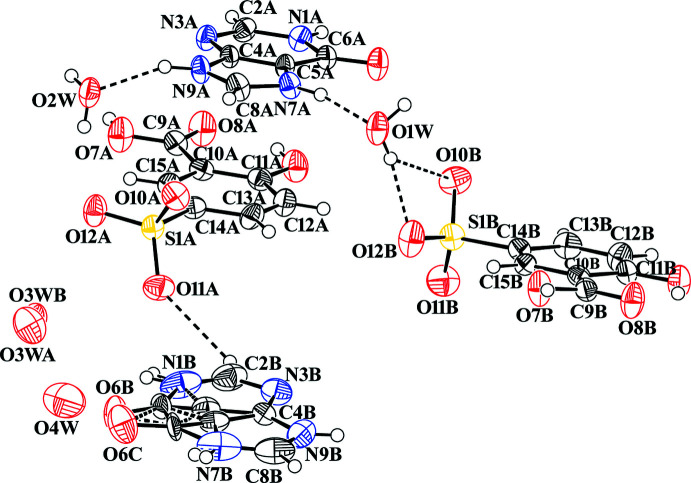
The asymmetric unit of salt (**I**), showing the atom-numbering scheme. Displacement ellipsoids are drawn at the 50% probability level. Dashed lines indicate hydrogen bonding and the disorder of cation B^+^ is shown.

Salt (**I**) crystallizes with two hypoxanthinium cations (A^+^ and B^+^), two 5-sufosalicylate anions (5SCA^−^; A and B) and four solvent water mol­ecules (O1*W*, O2*W*, O3W and O4*W*) in the asymmetric unit, as shown in Fig. 1 ▸. In salt (**I**), the B cation is equally disordered over two sets of sites for atoms C5*B*/C5*C*, C6*B*/C6*C* and O6*B*/O6*C*. Atoms H1*B*/H1*C* and H7*B*/H7*C* attached to N1*B* and N7*B*, respectively, are also disordered. The solvent water mol­ecule O3*W* is also disordered over two positions. Atoms N7*A* and N7*B* are protonated, which is confirmed by widening of the C5*A*—N7*A*—C8*A* angle to 107.1 (4)° compared to the value of 103.8° in the two polymorphic forms of the neutral HX mol­ecule (Schmalle *et al.*, 1988 ▸; Yang & Xie, 2007 ▸); the situation for C5*B*—N7*B*—C8*B* is less clear due to the observed disorder. The torsion angles of N3*A*—C4*A*—C5*A*—N7*A* = −179.2 (4)° and N3*B*—C4*B*—C5*C*—N7*B* = −178.3 (6)° are similar to those of the two forms of the neutral HX mol­ecule (−179.55 and −178.99°; Schmalle *et al.*, 1988 ▸; Yang & Xie, 2007 ▸). The carb­oxy­lic acid group in each of the two 5SCA^−^ anions is coplanar with the benzene ring [O7*A*—C9*A*—C10*A*—C11*A* = −178.2 (4)° and O7*B*—C9*B*—C10*B*—C11*B* = 175.9 (4)°], a situation that is likewise observed for previously reported crystal structures involving 5SCA^−^ anions. 
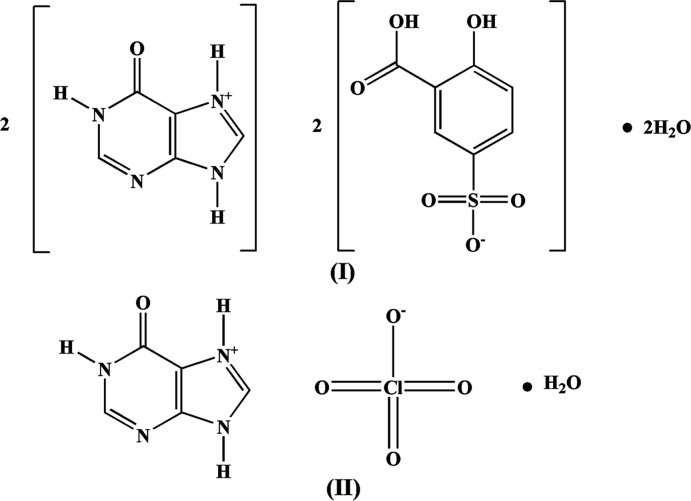


**Figure 2:**
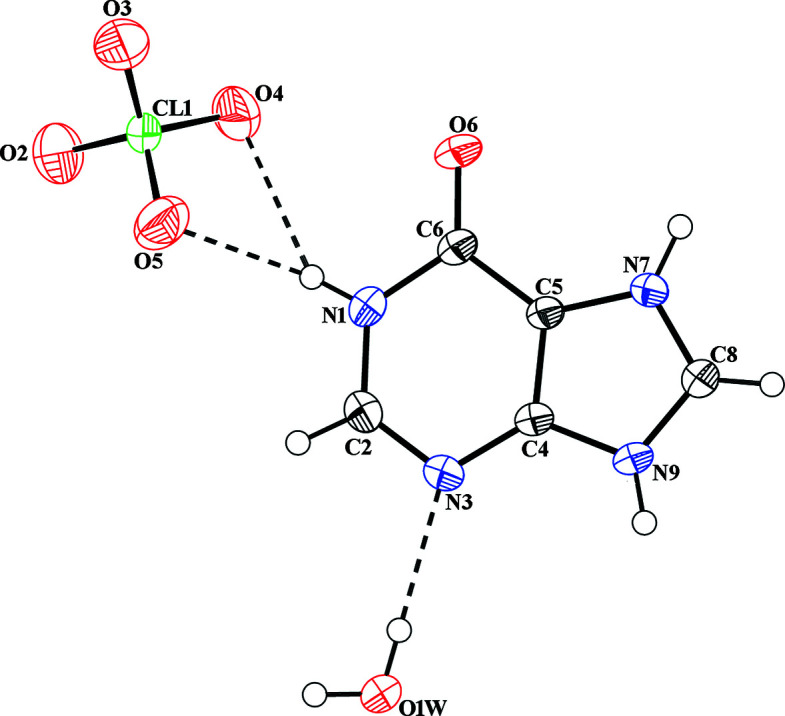
The asymmetric unit of salt (**II**), showing the atom-numbering scheme. Displacement ellipsoids are drawn at the 50% probability level. Dashed lines indicate hydrogen bonding.

Salt (**II**) crystallizes with one hypoxanthinium cation, one perchlorate anion (PCA^−^) and one solvent water mol­ecule in the asymmetric unit. The mol­ecular structure of salt (**II**) is shown in Fig. 2 ▸. Again, the N7 atom of the purine ring is protonated, as confirmed by the widening of the C5—N7—C8 angle to 108.00 (12)°. The N3—C4—C5—N7 torsion angle of 179.34 (14)° is similar to the values determined for salt (**I**). The PCA^−^ anion has the characteristic tetra­hedral shape, with Cl—O bond lengths between 1.4116 (15) and 1.4421 (15) Å, and O—Cl—O angles between 108.29 (9) and 111.24 (12)°.

## 3. Supra­molecular features

**Figure 3:**
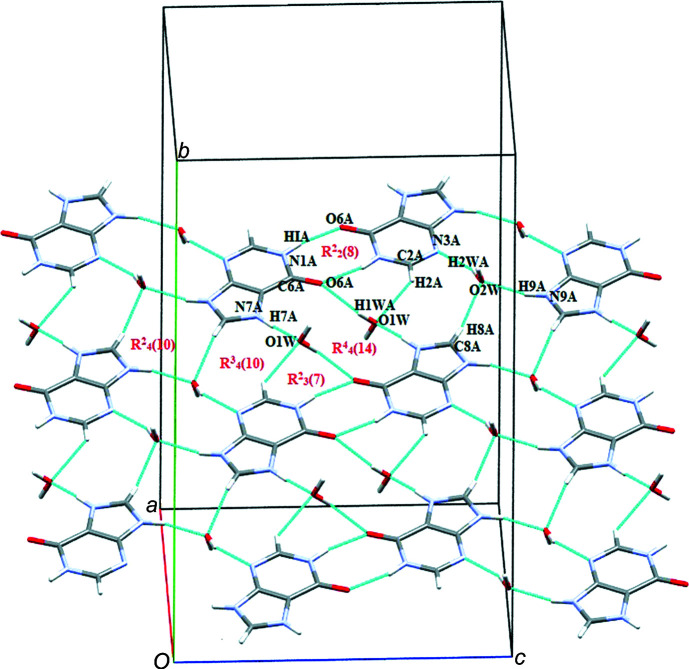
The crystal packing of (**I**), showing the N—H⋯O and O—H⋯O ring motifs formed between the A^+^ cation and water mol­ecules.

In the crystal structure of salt (**I**), (010) sheets of cations and sheets of anions are stacked alternately along [010]. The crystal packing is governed by N—H⋯O, O—H⋯N and C—H⋯O hydrogen bonds (Table 1 ▸). Symmetry-related A^+^ cations inter­act through a pair of N1*A*—H1*A*⋯O6*A* hydrogen bonds with a robust 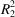 (8) motif (Bernstein *et al.*, 1995 ▸; Motherwell *et al.*, 2000 ▸). Solvent water mol­ecule O*W*1 connects the A^+^ cation *via* N7*A*—H7*A*⋯O1*W* and O1*W*—H1*WA*⋯O6*A* hydrogen bonds with an 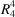 (14) motif. The A^+^ cations are further connected *via* C2*A*—H2*A*⋯O1*W*, C8*A*—H8*A*⋯O2*W*, N9*A*—H9*A*⋯O2*W* and O2*W*—H2*WA*⋯N3*A*, N1*A*—H1*A*⋯O6*A* hydrogen bonds with 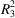 (7), 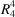 (14), 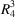 (10) and 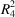 (10) motifs (Fig. 3 ▸).

**Figure 4:**
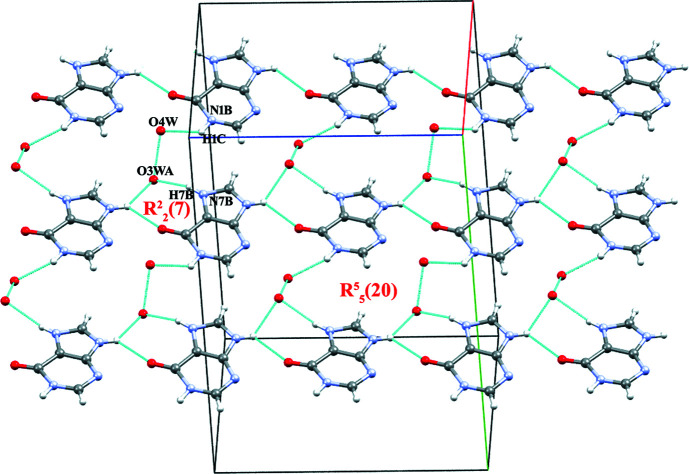
The crystal packing of (**I**), showing the N—H⋯O and O—H⋯O ring motifs formed between the B^+^ cation and (disordered) water mol­ecules.

**Figure 5:**
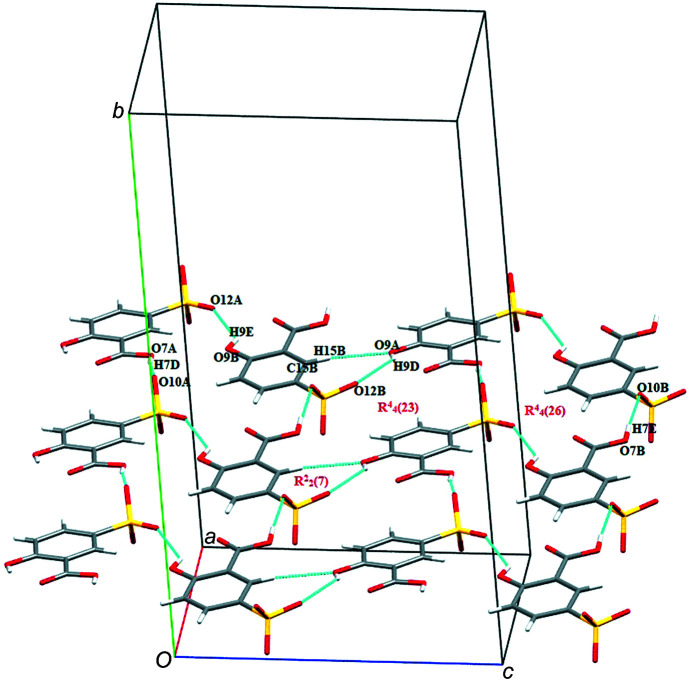
The supra­molecular layer of assembled 5SCA^−^ anions in salt (**I**).

**Figure 6:**
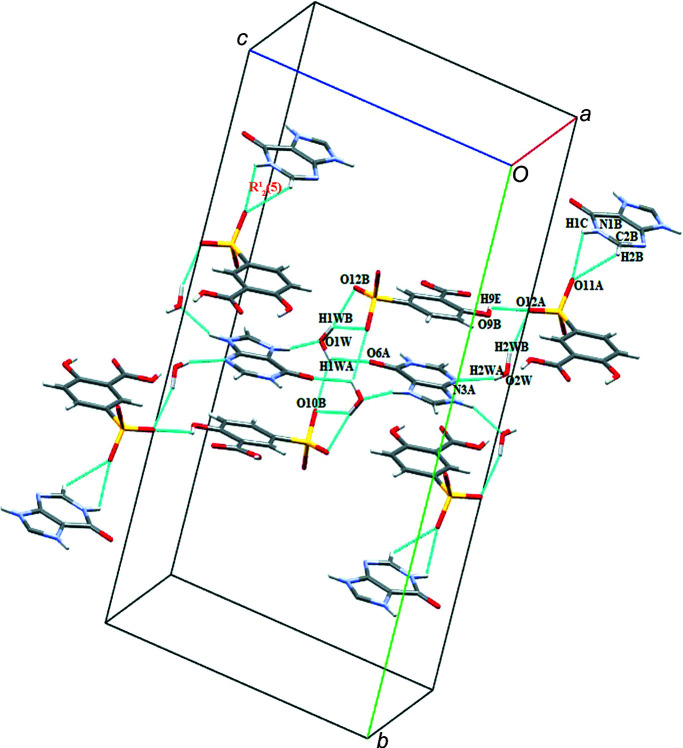
The alternating arrangement of cationic and anionic sheets in salt (**I**).

The B^+^ cations inter­act with the O atom of the solvent water mol­ecules O3*W* and O4*W* through N1*B*—H1*C*⋯O4*W* and N9*B*—H9*B*⋯O3*WA*, and with N9*B*—H9*B*⋯O6*B* with an 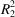 (7) motif. Short O3*WA*⋯O4*W* contacts with an 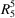 (20) motif are also observed (Fig. 4 ▸). Furthermore, the two 5SCA^−^ anions (A and B) self assemble into sheets by inter­action of symmetry-related counterparts through O7*A*—H7*D*⋯O10*A* and O7*B*—H7*E*⋯O10*B*, respectively (Fig. 5 ▸). A and B sheets are inter­connected through O9*B*—H9*E*⋯O12*A* and through O9*A*—H9*D*⋯O12*B* and C15*B*—H15*B*⋯O9*A* inter­actions, resulting in 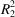 (7), 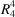 (23) and 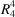 (26) ring motifs. Moreover, cation B^+^ inter­acts with 5SCA^−^ (A) *via* N1*B*—H1*C*⋯O11*A* and C2*B*—H2*B*⋯O11*A* with an 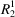 (5) motif. Another inter­connection between cationic and anionic sheets involves the solvent water mol­ecules through O1*W*—H1*WA*⋯O10*B*, O1*W*—H1*WB*⋯O12*B*, O2*W*—H2*WA*⋯N3*A* and O2*W*—H2*WB*⋯O12*A* (Fig. 6 ▸).

**Figure 7:**
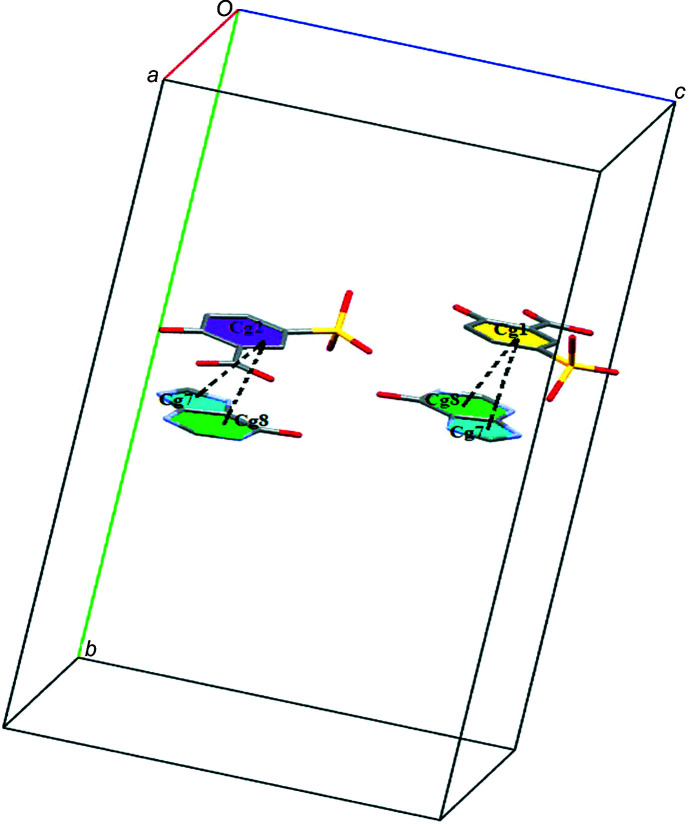
π–π stacking inter­actions in (**I**) between the imidazole and pyrimidine rings of the cations and the phenyl rings of the anions.

The crystal structure of (**I**) is consolidated by π–π inter­actions between the phenyl rings of the two 5SCA anions (C10*A*–C15*A* and C10*B*–C15*B*), and the imidazole ring (C4*A*–N9*A*) and the pyrimidine ring (N1*A*–C6*A*) of cation A^+^, with centroid-to-centroid distances of 3.547 (3), 3.562 (3), 3.554 (3) and 3.533 (3) Å, and slippages of 0.815, 1.300, 1.182 and 1.105 Å (Fig. 7 ▸).

**Figure 8:**
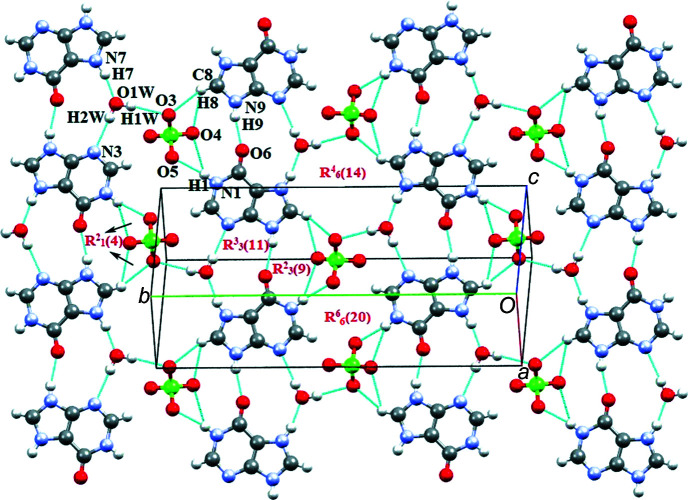
A view of the supra­molecular arrangement involving hydrogen-bonded rings in salt (**II**).

**Figure 9:**
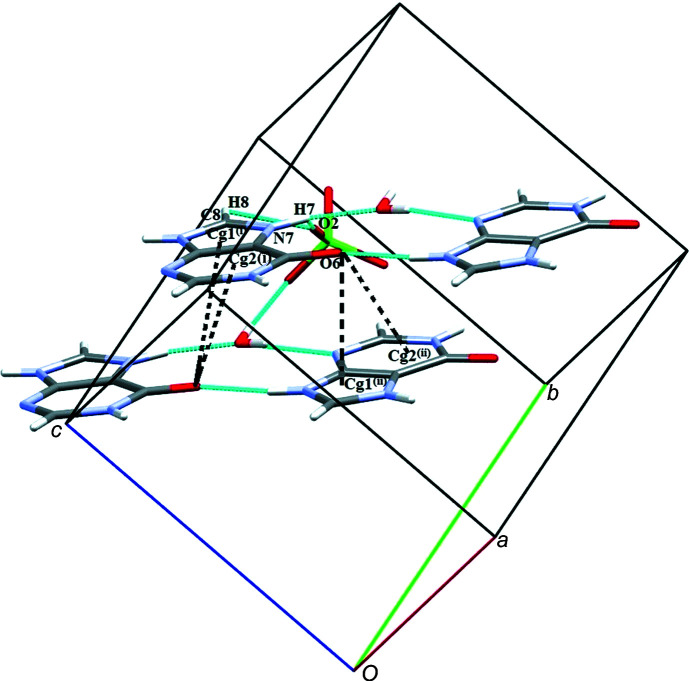
A view of the PCA^−^ anions and water mol­ecules connecting sheets through O—H⋯O hydrogen bonds and a view of the C—O⋯π inter­actions (π = imidazole and pyrimidine rings of the cation) in salt (**II**). [Symmetry codes: (i) −*x* + 2, *y* − 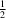 , −*z* + 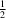 ; (ii) *x* + 1, −*y* + 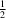 , *z* − 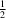 .]

In the crystal structure of salt (**II**), (010) sheets of cations and sheets of anions are stacked alternately along [010]. The crystal packing of salt (**II**) is dominated by N—H⋯O and O—H⋯O hydrogen bonds, and to a minor extent by C—H⋯O hydrogen bonds (Table 2 ▸). The protonated N atom of the cation forms an N7—H7⋯O1*W* ^ii^ hydrogen bond with the O atom of the water mol­ecule. The water mol­ecule disrupts the formation of base pairs but connects symmetry-related cations through O1*W*—H2*W*⋯N3^iv^. Additional N9—H9⋯O6^iii^ inter­actions with an 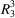 (11) ring motif generate a cationic strand along [201]. Parallel cationic strands are connected through the solvent water mol­ecule and the PCA^−^ anion through O1*W*—H1*W*⋯O3 and bifurcated N1—H1⋯O4 and N1—H1⋯O5 inter­actions, respectively, forming 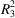 (9), 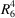 (14) and 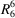 (20) motifs. The crystal packing of salt (**II**) is shown in Fig. 8 ▸. The crystal structure is further stabilized by carbon­yl⋯π (π refers to the ring system of the cation) inter­actions, with distances of 3.6097 (13), 3.2983 (13), 3.4580 (13) and 3.7236 (14) Å (Fig. 9 ▸).

## 4. Hirshfeld surface analysis

**Figure 10:**
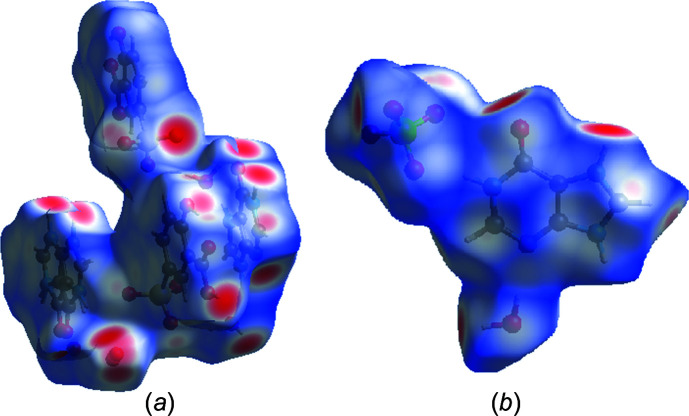
Hirshfeld surface for salts (*a*) (**I**) and (*b*) (**II**) mapped over *d*_norm_.

**Figure 11:**
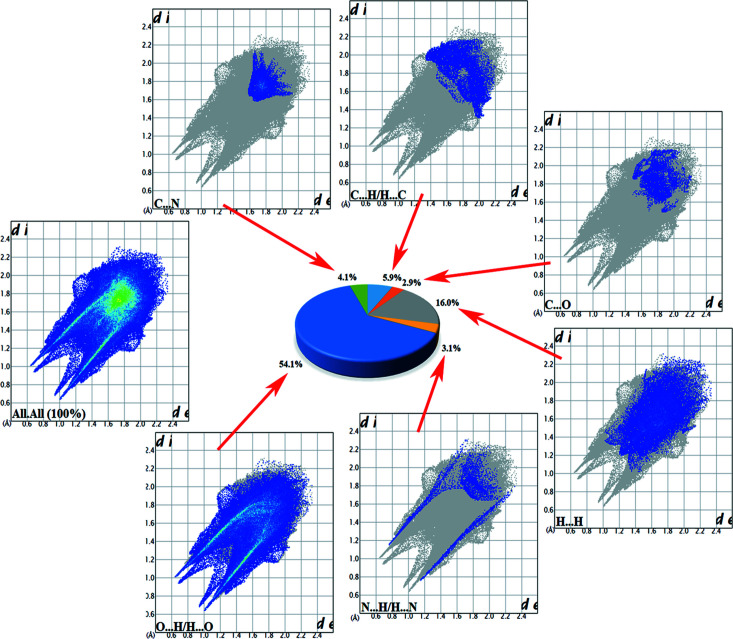
Fingerprint plots of salt (**I**) showing all inter­molecular inter­actions and delineated into O⋯H/H⋯O, H⋯N/N⋯H, C⋯O, C⋯N, C⋯H/H⋯C and H⋯H contacts.

Hirshfeld surface (HS) analyses of salts (**I**) and (**II**) were performed using *CrystalExplorer17* (Turner *et al.*, 2017 ▸). The results of the HS analysis mapped over *d*_norm_ are shown in Figs. 10 ▸(*a*) and 10(*b*) for (**I**) and (**II**), respectively. Corresponding two-dimensional fingerprint plots (Spackman & Jayatilaka, 2009 ▸) for (**I**) and (**II**) are shown in Figs. 11 ▸ and 12 ▸, respectively. The contributions of the noncovalent inter­actions to the HS in the two salts are: O⋯H/H⋯O 54.1% (**I**), 62.3% (**II**); N⋯H/H⋯N 3.1% (**I**), 6.8% (**II**); C⋯H/H⋯C 5.9% (**I**), 5.4% (**II**); H⋯H/H⋯H 16.0% (**I**), 5.3% (**II**); C⋯C/C⋯C 0.9% (**I**), 0.1% (**II**).

## 5. Comparison with the structures of related compounds

Crystal data, supra­molecular inter­actions and hydrogen bonding motifs of structurally similar halide/nitrate/phosphite/phosphate/perchlorate or sulfate salts like guanidinium bro­mide (Wei, 1977 ▸), guanidinium chloride (Maixner & Zachová, 1991 ▸), bis­(guanidinium) hydrogen phosphate 2.5-hydrate (Low *et al.*, 1986 ▸), guanidinium phosphite (Bendeif *et al.*, 2007 ▸), guanidinium sulfate (Cherouana *et al.*, 2003 ▸), guanidinium dinitrate dihydrate (Bouchouit *et al.*, 2002 ▸), xanthinium nitrate, xanthinium sulfate (Sridhar, 2011 ▸), xanthinium perchlorate dihydrate (Biradha *et al.*, 2010 ▸), hypoxanthinium chloride monohydrate (Sletten & Jensen, 1969 ▸) and hypoxanthinium nitrate monohydrate (Cabaj *et al.*, 2019 ▸) are listed and compared in Table 3 ▸.

**Figure 13:**
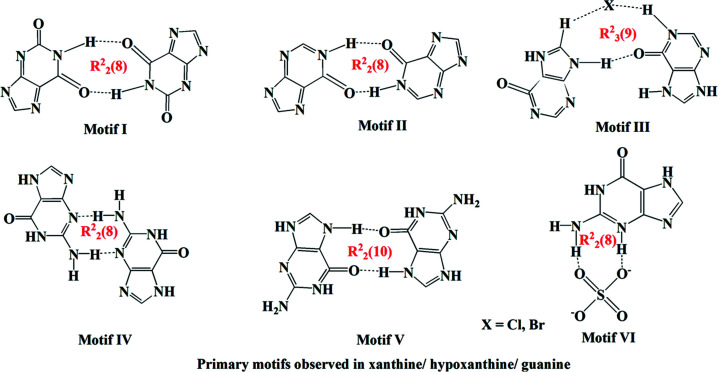
Primary ring motifs observed in purine derivatives.

A comparison of salts (**I**) and (**II**) with the related salt forms of guanine, xanthinium and hypoxanthine reveal that, in most of the crystal structures containing purine derivatives, the purine forms base pairs through pairs of N—H⋯O or N—H⋯N hydrogen bonds with an 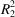 (8) primary ring motif. When it comes to an inter­action between the purine base and a strong acid, the chloride/nitrate/sulfate/phosphite/phosphate or perchlorate salts of guanine/xanthine and hypoxanthine have different mol­ecular recognition patterns. The most important primary and secondary motifs formed by hypoxanthine and similar compounds are summarized in Figs. 13 ▸ and 14 ▸. Crystallographic studies of salts involving perchlorate and sulfate anions reveal that most of these salts have similar crystal packing arrangements (Bishop *et al.*, 2014 ▸). In general, salts of structurally similar systems will have similar mol­ecular recognition patterns and supra­molecular motifs. However, for salts (**I**) and (**II**) and related systems compiled in Table 3 ▸, great similarities are not observed. The differences in mol­ecular recognition and supra­molecular self-assembly might be due to the involvement of other functional groups or substituents in the structures, the intrusion of water mol­ecules in the crystal structure, or the ratio of anions and cations present in the asymmetric unit.

## 6. Synthesis and crystallization

Salt (**I**) was synthesized by mixing an equimolar ratio of hypoxanthine (0.0340 g) and 5-sulfosalicylic acid (0.0545 g) in hot water. The solution was heated to 333 K for 1 h and then allowed to cool slowly to room tem­per­ature. Colourless needle-shaped crystals were harvested from the mother liquid after one week.

Salt (**II**) was synthesized by mixing an equimolar ratio of hypoxanthine (0.0340 g) and iron perchlorate monohydrate (0.0681 g) in hot water. The solution was heated to 333 K with constant stirring for 1 h and then allowed to cool slowly to room tem­per­ature. Colourless plate-like crystals were harvested from the mother liquid after one week.

## 7. Refinement

Crystal data, data collection and structure refinement details of salts (**I**) and (**II**) are summarized in Table 4 ▸. In salt (**I**), carbon (C5 and C6) and oxygen (O6) atoms of cation B are equally disordered over two sets of sites, with a refined occupancy ratio of 0.503 (18):0.497 (18). The solvent water mol­ecule O3*W* is disordered over two positions, with a refined site-occupancy ratio of 0.58 (6):0.42 (6). The H atoms of water mol­ecules O1*W* and O2*W* were located from a difference Fourier map, and the O—H distance restrained to 0.82 Å. Attempts to localize the H atoms of O3*W* and O4*W* in (**I**) from difference Fourier maps failed as there were no relevant electron densities close to these atoms. Hence, these H atoms are not part of the model but are included in the formula. All C- and N-bound H atoms in (**I**) were placed in idealized positions and refined freely using a riding model, with C—H = 0.95 Å and N—H = 0.86 Å, and with *U*_iso_(H) = 1.2*U*_eq_(C,N). In salt (**II**), the N-bound H atoms were located in a difference Fourier map and refined freely. The H atoms of the water mol­ecule were likewise located from a difference Fourier map. The geometry of the water mol­ecule was restrained using DFIX commands with an O—H distance of 0.85 Å and an H⋯H distance of 1.36 Å. All C-bound H atoms were treated as for salt (**I**).

## Supplementary Material

Crystal structure: contains datablock(s) I, II, global. DOI: 10.1107/S2056989022004753/wm5640sup1.cif

Structure factors: contains datablock(s) I. DOI: 10.1107/S2056989022004753/wm5640Isup2.hkl

Structure factors: contains datablock(s) II. DOI: 10.1107/S2056989022004753/wm5640IIsup3.hkl

CCDC references: 2170315, 2170314

Additional supporting information:  crystallographic information; 3D view; checkCIF report

## Figures and Tables

**Figure 12:**
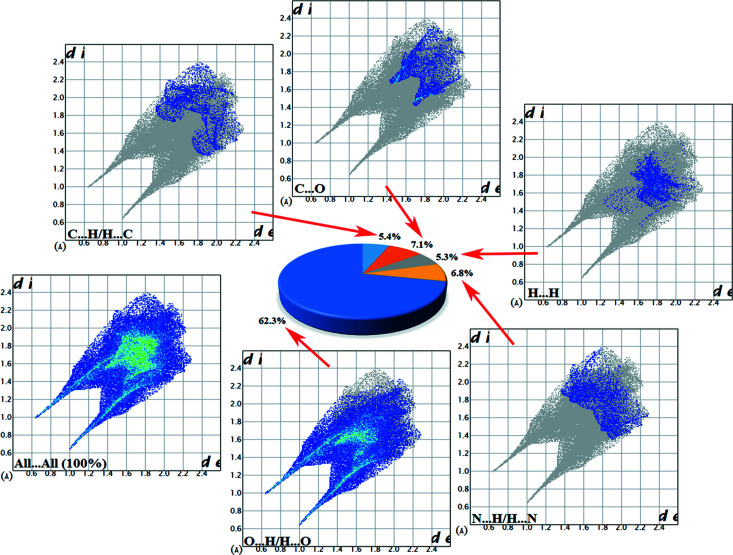
Fingerprint plots of salt (**II**) showing all inter­molecular inter­actions and delineated into O⋯H/H⋯O, H⋯N/N⋯H, C⋯O, C⋯H/H⋯C and H⋯H contacts.

**Figure 14:**
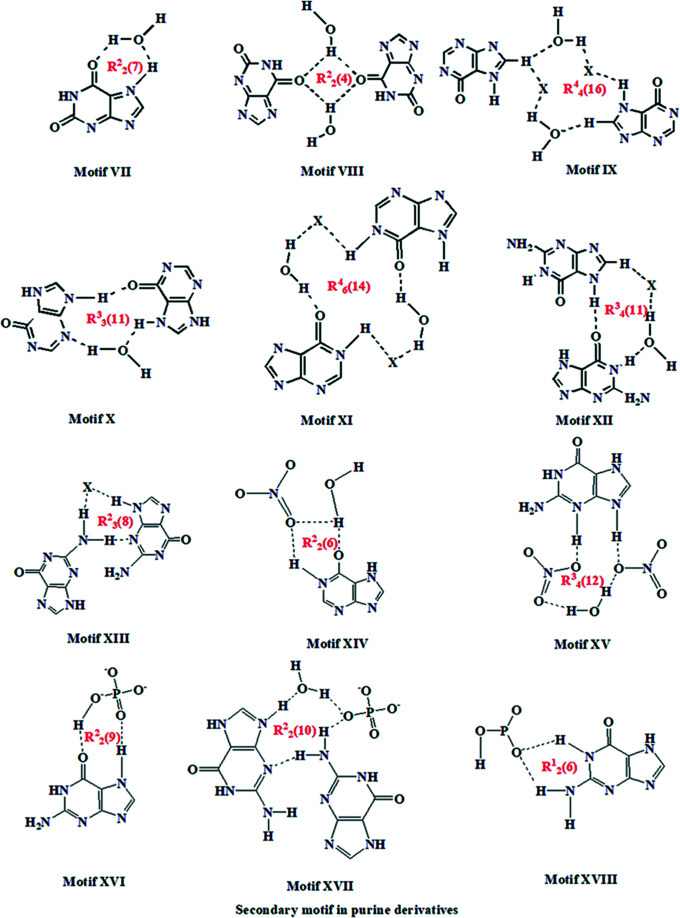
Secondary ring motifs observed in purine derivatives.

**Table 1:** Hydrogen-bond geometry (Å, °) for (**I**)

*D*—H⋯*A*	*D*—H	H⋯*A*	*D*⋯*A*	*D*—H⋯*A*
N7*B*—H7*B*⋯O3*WA* ^i^	0.86	2.26	3.08	158
O7*A*—H7*D*⋯O10*A* ^ii^	0.82	1.86	2.677	170
O7*B*—H7*E*⋯O10*B* ^i^	0.82	1.84	2.655	175
O9*A*—H9*D*⋯O12*B* ^ii^	0.82	2.34	2.924	128
O9*B*—H9*E*⋯O12*A* ^iii^	0.82	2.54	3.143	131
O1*W*—H1*WA*⋯O6*A* ^iv^	0.85	2.31	2.801	117
O1*W*—H1*WA*⋯O10*B* ^iv^	0.85	2.28	2.917	132
N9*B*—H9*B*⋯O6*B* ^v^	0.86	2.42	3.044	130
N9*B*—H9*B*⋯O3*WA* ^vi^	0.86	2.47	3.07	128
N1*A*—H1*A*⋯O6*A* ^vii^	0.86	2.05	2.898	170
N1*B*—H1*C*⋯O4*W*	0.86	2.22	2.890	135
N1*B*—H1*C*⋯O11*A*	0.86	2.45	2.998	122
O1*W*—H1*WB*⋯O12*B*	0.85	2.01	2.844	169
O2*W*—H2*WA*⋯N3*A*	0.83	2.07	2.849	157
O2*W*—H2*WB*⋯O12*A*	0.82	2.03	2.815	160
N7*A*—H7*A*⋯O1*W*	0.86	1.77	2.615	168
N9*A*—H9*A*⋯O2*W*	0.86	1.89	2.697	157
C2*A*—H2*A*⋯O1*W* ^ii^	0.93	2.43	3.149	134
C2*B*—H2*B*⋯O11*A*	0.93	2.46	2.974	114
C8*A*—H8*A*⋯O2*W* ^viii^	0.93	2.40	3.310	167
C15*B*—H15*B*⋯O9*A*	0.93	2.59	3.510	172

Symmetry codes: (i) 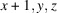 ; (ii) 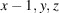 ; (iii) 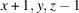 ; (iv) 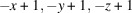 ; (v) 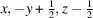 ; (vi) 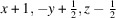 ; (vii) 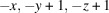 ; (viii) 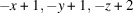 .

**Table 2:** Hydrogen-bond geometry (Å, °) for (**II**)

*D*—H⋯*A*	*D*—H	H⋯*A*	*D*⋯*A*	*D*—H⋯*A*
N1—H1⋯O4	0.82	2.60	3.249	138
N1—H1⋯O5	0.82	2.09	2.879	162
N7—H7⋯O2^i^	0.91	2.60	3.031	110.2
N7—H7⋯O1*W* ^ii^	0.91	1.76	2.6489	165
N9—H9⋯O6^iii^	0.84	1.93	2.7602	166
O1*W*—H1*W*⋯O3^iv^	0.85	2.17	3.018	172
O1*W*—H2*W*⋯N3	0.85	2.11	2.951	172
C8—H8⋯O2^i^	0.93	2.47	2.970	114
C8—H8⋯O3^iii^	0.93	2.47	3.268	144
C8—H8⋯O4^iii^	0.93	2.55	3.072	116

Symmetry codes: (i) 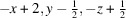 ; (ii) 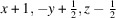 ; (iii) 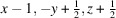 ; (iv) 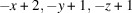 .

**Table 3:** Comparison of salt forms of purine derivatives containing halides/nitrate/phosphite/phosphate/sulfate and perchlorates as anions

Compound	Space group	Primary inter­action between	Graph-set motif	Motif type	Secondary inter­action between	Graph-set motif	Motif type	
Guanidinium hydro­chloride	Monoclinic *P*2_1_/*c*,	N—H⋯N,	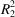 (8),	IV and V	N—H⋯Cl,	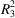 (8),	XII and XIII	
	*a* = 4.479 Å	N—H⋯O	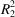 (10)		C—H⋯Cl,	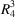 (11)		
	*b* = 9.995 Å				O—H⋯N,			
	*c* = 19.304 Å				O—H⋯Cl			
	β = 107.90°							
Guanidinium hydro­bromide	Monoclinic *P*2_1_/*c*	N—H⋯N,	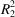 (8),	IV and V	N—H⋯Br,	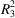 (8),	XII and XIII	
	*a* = 4.8708 Å	N—H⋯O	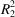 (10)		N—H⋯N,	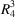 (11)		
	*b* = 13.237 Å				O—H⋯Br,			
	*c* = 14.638 Å				C—H⋯Br			
	β = 93.906°							
Guanidinium dinitrate dihydrate	Monoclinic *P*2_1_/*c*	N—H⋯O	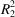 (8)	V	N—H⋯O,	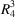 (12)	XII	
	*a* = 6.6340 Å				O—H⋯O			
	*b* = 10.2020 Å							
	*c* = 11.0440 Å							
	β = 106.04°							
Guanidinium phosphite monohydrate	Monoclinic *P*2_1_/*c*	N—H⋯N	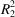 (8)	IV	N—H⋯O	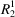 (6),	XII and XVIII	
	*a* = 4.9700 Å					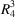 (10)		
	*b* = 12.7506 Å							
	*c* = 15.0499 Å							
	β = 92.293°							
Guanidinium phosphite dihydrate form (I)	Monoclinic *P*2_1_/*c*	N—H⋯N	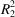 (8)	IV	N—H⋯N,	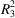 (8),	XIII and XVIII	
	*a* = 4.6812 Å				N—H⋯O	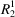 (6)		
	*b* = 24.0561 Å							
	*c* = 9.5186 Å							
	β = 99.773°							
Guanidinium phosphite dihydrate form (II)	Monoclinic *P*2_1_/*c*	N—H⋯N	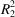 (8)	IV	N—H⋯N,	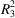 (8),	XIII and XVIII	
	*a* = 4.7340 Å				N—H⋯O	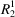 (6)		
	*b* = 24.0450 Å							
	*c* = 9.5050 Å							
	β = 98.860°							
Guanidinium phosphate hydrate form (I)	Triclinic, *P* 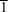	N—H⋯N	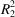 (8)	IV	N—H⋯O,	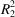 (9)	XVI and XVII	
	*a* = 9.607 Å				O—H⋯O	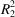 (10)		
	*b* = 10.221 Å							
	*c* = 10.603 Å							
	α = 84.5°							
	β = 108.2°							
	γ = 119.7°							
Guanidinium phosphate monohydrate form (II)	Monoclinic *P*2_1_/*n*	N—H⋯N	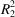 (8)	IV	N—H⋯O,	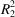 (8),	VI, XIII and XVI	
	*a* = 4.5414 Å				O—H⋯O	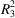 (8),		
	*b* = 12.5774 Å					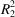 (9)		
	*c* = 18.1485 Å							
	β = 93.689 °							
Guanidinium sulfate monohydrate	Monoclinic *P*2_1_/*c*	N—H⋯O	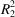 (8)	VI	N—H⋯O,	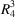 (12)	XV	
	*a* = 8.9940 Å				O—H⋯O			
	*b* = 10.2020 Å							
	*c* = 11.0440 Å							
	β = 106.04°							
Xanthinium nitrate monohydrate	Triclinic, *P* 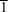	N—H⋯O	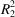 (8)	I	O—H⋯N,	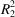 (4),	VIII, XI and XIII	
	*a* = 5.0416 Å				O—H⋯O	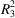 (8),		
	*b* = 7.4621 Å					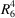 (14)		
	*c* = 12.1396 Å							
	α = 80.248°							
	β = 80.800°							
	γ = 75.657°							
Xanthinium sulfate monohydrate	Monoclinic *P*2_1_	N—H⋯O	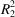 (8)	I	O—H⋯N,	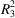 (8)	XIII	
	*a* = 5.183 Å							
	*b* = 24.805 Å							
	*c* = 7.701 Å							
	β = 103.510°							
Xanthinium perchlorate dihydrate	Triclinic, *P* 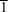	N—H⋯O	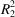 (8)	I	O—H⋯N,	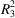 (8)	XIII	
	*a* = 5.1625 Å				O—H⋯O			
	*b* = 7.7449 Å							
	*c* = 13.696 Å							
	α = 100.214°							
	β = 91.591°							
	γ = 100.880°							
Hypoxanthinium hydro­chloride monohydrate	Monoclinic *P*2_1_/*c*	N—H⋯Cl	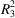 (9)	III	N—H⋯Cl,	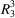 (11),	IX, X and XI	
	*a* = 4.8295 Å				C—H⋯Cl,	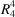 (16),		
	*b* = 17.7285 Å				O—H⋯N,	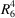 (14)		
	*c* = 9.0077 Å				O—H⋯Cl			
	β = 94.59°							
Hypoxanthinium nitrate monohydrate form (I)	Ortho­rhom­bic *Pnma*	N—H⋯O	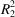 (8)	II	N—H⋯O,	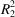 (6),	XIII and XIV	
	*a* = 13.701 Å				O—H⋯O,	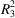 (8),		
	*b* = 6.236 Å					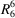 (20)		
	*c* = 10.078 Å							
Hypoxanthinium nitrate monohydrate form (II)	Monoclinic *P*2_1_/*n*	N—H⋯O	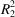 (8)	II	N—H⋯O,	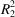 (6),	XIII and XIV	
	*a* = 6.1452 Å				O—H⋯O,	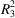 (8)		
	*b* = 13.7517 Å							
	*c* = 10.0414 Å							
	β = 95.601°							

**Table 4:** Experimental details

	(I)	(II)
Crystal data		
Chemical formula	C_5_H_5_N_4_O^+^·C_7_H_5_O_6_S^−^·2H_2_O	C_5_H_5_N_4_O^+^·ClO_4_^−^·H_2_O
*M*_r_	388.32	254.60
Crystal system, space group	Monoclinic, *P*2_1_/*c*	Monoclinic, *P*2_1_/*c*
Temperature (K)	293	296
*a*, *b*, *c* (Å)	8.7055 (3), 25.9927 (13), 13.6479 (5)	5.0307 (6), 20.386 (2), 9.0181 (10)
β (°)	91.864 (3)	94.233 (2)
*V* (Å^3^)	3086.6 (2)	922.33 (18)
*Z*	8	4
Radiation type	Mo *K*α	Mo *K*α
μ (mm^−1^)	0.27	0.44
Crystal size (mm)	0.55 × 0.20 × 0.10	0.45 × 0.02 × 0.003

Data collection		
Diffractometer	Bruker APEXII CCD	Bruker APEXII CCD
Absorption correction	–	Multi-scan (*SADABS*; Bruker, 2016 ▸)
*T*_min_, *T*_max_	–	0.957, 1.000
No. of measured, independent and observed [*I* > 2σ(*I*)] reflections	21075, 7079, 5905	16360, 2752, 2370
*R*_int_	0.032	0.025
(sin θ/λ)_max_ (Å^−1^)	0.649	0.711

Refinement		
*R*[*F* ^2^ > 2σ(*F* ^2^)], *wR*(*F* ^2^), *S*	0.087, 0.190, 1.22	0.038, 0.111, 1.05
No. of reflections	7079	2752
No. of parameters	521	165
No. of restraints	2	3
H-atom treatment	H atoms treated by a mixture of independent and constrained refinement	H atoms treated by a mixture of independent and constrained refinement
Δρ_max_, Δρ_min_ (e Å^−3^)	0.74, −0.43	0.37, −0.29

Computer programs: *APEX2* and *SAINT* (Bruker, 2016 ▸), *SHELXT* (Sheldrick, 2015*a* ▸), *SHELXL* (Sheldrick, 2015*b* ▸), *Mercury* (Macrae *et al.*, 2020 ▸), *POVRay* (Cason, 2004 ▸), *PLATON* (Spek, 2020 ▸) and *publCIF* (Westrip, 2010 ▸).

## supplementary crystallographic information

### 6-Oxo-1,9-dihydropurin-7-ium 5-sulfosalicylate dihydrate (I) Crystal data

**Table:**

C_5_H_5_N_4_O^+^·C_7_H_5_O_6_S^−^·2H_2_O	*F*(000) = 1600
*M**_r_* = 388.32	*D*_x_ = 1.671 Mg m^−^^3^
Monoclinic, *P*2_1_/*c*	Mo *K*α radiation, λ = 0.71073 Å
*a* = 8.7055 (3) Å	Cell parameters from 7079 reflections
*b* = 25.9927 (13) Å	θ = 2.8–27.5°
*c* = 13.6479 (5) Å	µ = 0.27 mm^−^^1^
β = 91.864 (3)°	*T* = 293 K
*V* = 3086.6 (2) Å^3^	Needle, colourless
*Z* = 8	0.55 × 0.20 × 0.10 mm

### 6-Oxo-1,9-dihydropurin-7-ium 5-sulfosalicylate dihydrate (I) Data collection

**Table:**

Bruker APEXII CCD diffractometer	*R*_int_ = 0.032
φ and ω scans	θ_max_ = 27.5°, θ_min_ = 2.9°
21075 measured reflections	*h* = −11→11
7079 independent reflections	*k* = −23→33
5905 reflections with *I* > 2σ(*I*)	*l* = −17→17

### 6-Oxo-1,9-dihydropurin-7-ium 5-sulfosalicylate dihydrate (I) Refinement

**Table:**

Refinement on *F*^2^	Hydrogen site location: mixed
Least-squares matrix: full	H atoms treated by a mixture of independent and constrained refinement
*R*[*F*^2^ > 2σ(*F*^2^)] = 0.087	*w* = 1/[σ^2^(*F*_o_^2^) + (0.0167*P*)^2^ + 13.1153*P*] where *P* = (*F*_o_^2^ + 2*F*_c_^2^)/3
*wR*(*F*^2^) = 0.190	(Δ/σ)_max_ < 0.001
*S* = 1.22	Δρ_max_ = 0.74 e Å^−^^3^
7079 reflections	Δρ_min_ = −0.43 e Å^−^^3^
521 parameters	Extinction correction: SHELXL (Sheldrick, 2015b), Fc^*^=kFc[1+0.001xFc^2^λ^3^/sin(2θ)]^-1/4^
2 restraints	Extinction coefficient: 0.0013 (3)

### 6-Oxo-1,9-dihydropurin-7-ium 5-sulfosalicylate dihydrate (I) Special details

**Table:**

Geometry. All esds (except the esd in the dihedral angle between two l.s. planes) are estimated using the full covariance matrix. The cell esds are taken into account individually in the estimation of esds in distances, angles and torsion angles; correlations between esds in cell parameters are only used when they are defined by crystal symmetry. An approximate (isotropic) treatment of cell esds is used for estimating esds involving l.s. planes.

### 6-Oxo-1,9-dihydropurin-7-ium 5-sulfosalicylate dihydrate (I) Fractional atomic coordinates and isotropic or equivalent isotropic displacement parameters (Å^2^)

**Table:**

	*x*	*y*	*z*	*U*_iso_*/*U*_eq_	Occ. (<1)
O6A	0.1947 (4)	0.49617 (15)	0.5394 (2)	0.0415 (8)	
N1A	−0.0096 (4)	0.49891 (15)	0.6407 (3)	0.0312 (8)	
H1A	−0.072122	0.497638	0.590694	0.037*	
N3A	0.0062 (4)	0.50335 (15)	0.8125 (3)	0.0311 (8)	
N7A	0.3886 (4)	0.49922 (15)	0.7338 (3)	0.0321 (8)	
H7A	0.459823	0.497846	0.691626	0.038*	
N9A	0.2725 (4)	0.50320 (16)	0.8730 (3)	0.0331 (9)	
H9A	0.257740	0.504829	0.934876	0.040*	
C2A	−0.0710 (5)	0.50158 (18)	0.7299 (3)	0.0325 (10)	
H2A	−0.177576	0.502197	0.732324	0.039*	
C4A	0.1607 (5)	0.50245 (17)	0.8000 (3)	0.0263 (9)	
C5A	0.2318 (4)	0.50003 (17)	0.7134 (3)	0.0261 (9)	
C6A	0.1473 (5)	0.49805 (18)	0.6231 (3)	0.0287 (9)	
C8A	0.4084 (5)	0.50097 (19)	0.8307 (3)	0.0355 (10)	
H8A	0.503178	0.500683	0.864222	0.043*	
N1B	0.7702 (5)	0.25698 (19)	0.9705 (5)	0.0620 (15)	
H1C	0.689866	0.258941	1.005445	0.074*	0.497 (18)
H1B	0.716586	0.258513	1.022312	0.074*	0.503 (18)
C2B	0.7345 (7)	0.2589 (2)	0.8774 (6)	0.0581 (16)	
H2B	0.631879	0.262454	0.857329	0.070*	
N3B	0.8390 (5)	0.25613 (17)	0.8117 (3)	0.0445 (10)	
C4B	0.9779 (5)	0.25070 (18)	0.8592 (3)	0.0331 (10)	
O6B	0.9419 (18)	0.2493 (6)	1.1190 (8)	0.054 (3)	0.497 (18)
C5C	1.0267 (15)	0.2496 (4)	0.9577 (8)	0.035 (3)	0.497 (18)
C6B	0.9094 (14)	0.2524 (4)	1.0314 (7)	0.035 (3)	0.497 (18)
O6C	1.0195 (19)	0.2484 (6)	1.1206 (10)	0.062 (3)	0.503 (18)
C5B	0.9336 (17)	0.2513 (4)	0.9552 (8)	0.040 (3)	0.503 (18)
C6C	1.0442 (17)	0.2479 (4)	1.0326 (7)	0.048 (4)	0.503 (18)
N7B	1.1875 (6)	0.2444 (2)	0.9780 (5)	0.0680 (16)	
H7B	1.241971	0.242864	1.031666	0.082*	0.497 (18)
H7C	1.267726	0.243141	1.016423	0.082*	0.503 (18)
C8B	1.2225 (7)	0.2428 (2)	0.8848 (6)	0.0565 (16)	
H8B	1.324295	0.239139	0.867383	0.068*	
N9B	1.1141 (5)	0.24629 (17)	0.8172 (4)	0.0479 (11)	
H9B	1.127857	0.245844	0.755081	0.058*	
O3WA	0.367 (4)	0.2714 (4)	1.1689 (17)	0.063 (5)	0.58 (6)
O3WB	0.296 (4)	0.2700 (6)	1.1468 (11)	0.043 (6)	0.42 (6)
S1A	0.40654 (12)	0.36757 (5)	0.96698 (8)	0.0300 (3)	
O7A	−0.1915 (4)	0.38972 (17)	0.9153 (3)	0.0473 (10)	
H7D	−0.284261	0.394380	0.919381	0.071*	
O8A	−0.2547 (4)	0.38674 (17)	0.7567 (3)	0.0489 (10)	
O9A	−0.0354 (4)	0.37374 (16)	0.6296 (2)	0.0453 (9)	
H9D	−0.123261	0.380142	0.645875	0.068*	
O10A	0.5153 (4)	0.40963 (14)	0.9514 (3)	0.0386 (8)	
O11A	0.4769 (4)	0.31756 (14)	0.9629 (3)	0.0467 (9)	
O12A	0.3208 (4)	0.37511 (15)	1.0554 (2)	0.0429 (9)	
C9A	−0.1574 (5)	0.38438 (18)	0.8225 (3)	0.0309 (9)	
C10A	0.0083 (5)	0.37720 (17)	0.8061 (3)	0.0295 (9)	
C11A	0.0590 (5)	0.37254 (18)	0.7101 (3)	0.0322 (10)	
C12A	0.2158 (5)	0.3673 (2)	0.6944 (4)	0.0381 (11)	
H12A	0.250192	0.364424	0.630852	0.046*	
C13A	0.3201 (5)	0.36641 (19)	0.7726 (3)	0.0352 (10)	
H13A	0.424567	0.363245	0.761467	0.042*	
C14A	0.2696 (5)	0.37022 (17)	0.8681 (3)	0.0299 (9)	
C15A	0.1145 (5)	0.37553 (17)	0.8842 (3)	0.0287 (9)	
H15A	0.080905	0.378003	0.948039	0.034*	
S1B	0.56102 (12)	0.37011 (5)	0.45829 (9)	0.0341 (3)	
O7B	1.1577 (4)	0.38612 (17)	0.4147 (2)	0.0443 (9)	
H7E	1.248391	0.394576	0.418788	0.066*	
O8B	1.2092 (4)	0.37883 (17)	0.2566 (3)	0.0497 (10)	
O9B	0.9832 (4)	0.36982 (17)	0.1295 (2)	0.0492 (9)	
H9E	1.073533	0.369136	0.148463	0.074*	
O10B	0.4520 (4)	0.41174 (15)	0.4380 (3)	0.0465 (9)	
O11B	0.4889 (4)	0.31994 (16)	0.4549 (3)	0.0542 (10)	
O12B	0.6496 (4)	0.37900 (17)	0.5491 (3)	0.0509 (10)	
C9B	1.1165 (5)	0.37948 (18)	0.3224 (3)	0.0317 (10)	
C10B	0.9504 (5)	0.37406 (17)	0.3041 (3)	0.0280 (9)	
C11B	0.8925 (5)	0.37007 (18)	0.2071 (3)	0.0333 (10)	
C12B	0.7338 (6)	0.3667 (2)	0.1889 (4)	0.0405 (11)	
H12B	0.695190	0.363737	0.124781	0.049*	
C13B	0.6350 (5)	0.36764 (19)	0.2646 (4)	0.0365 (11)	
H13B	0.529554	0.365968	0.251642	0.044*	
C14B	0.6914 (5)	0.37113 (17)	0.3618 (3)	0.0287 (9)	
C15B	0.8480 (5)	0.37456 (16)	0.3811 (3)	0.0269 (9)	
H15B	0.885372	0.377214	0.445531	0.032*	
O1W	0.6140 (4)	0.48175 (16)	0.6162 (2)	0.0449 (9)	
H1WA	0.605842	0.503025	0.569001	0.067*	
H1WB	0.613001	0.452409	0.588798	0.067*	
O2W	0.2476 (4)	0.48039 (15)	1.0649 (3)	0.0412 (8)	
O4W	0.6239 (8)	0.2277 (2)	1.1488 (4)	0.100 (2)	
H2WA	0.161 (5)	0.487 (4)	1.082 (7)	0.150*	
H2WB	0.270 (12)	0.4502 (13)	1.075 (8)	0.150*	

### 6-Oxo-1,9-dihydropurin-7-ium 5-sulfosalicylate dihydrate (I) Atomic displacement parameters (Å^2^)

**Table:**

	*U*^11^	*U*^22^	*U*^33^	*U*^12^	*U*^13^	*U*^23^
O6A	0.0336 (17)	0.066 (2)	0.0244 (16)	−0.0003 (17)	0.0008 (13)	−0.0022 (16)
N1A	0.0239 (18)	0.041 (2)	0.0279 (19)	0.0016 (16)	−0.0058 (14)	−0.0027 (17)
N3A	0.0270 (18)	0.040 (2)	0.0268 (19)	0.0015 (16)	0.0048 (15)	0.0003 (16)
N7A	0.0220 (17)	0.045 (2)	0.0296 (19)	0.0007 (16)	0.0021 (14)	0.0066 (17)
N9A	0.0300 (19)	0.049 (2)	0.0205 (17)	0.0018 (17)	−0.0007 (14)	0.0037 (17)
C2A	0.026 (2)	0.036 (2)	0.036 (2)	0.0014 (18)	0.0062 (18)	−0.002 (2)
C4A	0.028 (2)	0.031 (2)	0.0198 (19)	0.0007 (17)	0.0007 (16)	0.0010 (17)
C5A	0.0191 (18)	0.036 (2)	0.023 (2)	0.0006 (17)	0.0023 (15)	0.0005 (18)
C6A	0.030 (2)	0.034 (2)	0.023 (2)	−0.0007 (18)	0.0008 (16)	0.0000 (18)
C8A	0.025 (2)	0.047 (3)	0.034 (2)	−0.003 (2)	0.0002 (18)	0.007 (2)
N1B	0.034 (2)	0.046 (3)	0.106 (5)	0.002 (2)	0.006 (3)	−0.012 (3)
C2B	0.046 (3)	0.044 (3)	0.086 (5)	0.003 (3)	0.017 (3)	−0.002 (3)
N3B	0.037 (2)	0.043 (2)	0.052 (3)	0.0017 (19)	−0.008 (2)	0.003 (2)
C4B	0.038 (2)	0.029 (2)	0.032 (2)	0.0005 (19)	0.0027 (19)	0.0034 (19)
O6B	0.058 (7)	0.077 (7)	0.026 (4)	−0.009 (7)	−0.003 (5)	−0.003 (4)
C5C	0.023 (6)	0.030 (5)	0.051 (7)	0.004 (4)	−0.005 (5)	−0.003 (4)
C6B	0.052 (8)	0.034 (5)	0.020 (5)	−0.003 (5)	−0.004 (4)	−0.001 (4)
O6C	0.061 (8)	0.081 (7)	0.044 (6)	−0.014 (8)	−0.012 (6)	0.009 (5)
C5B	0.040 (8)	0.031 (5)	0.048 (7)	−0.006 (5)	0.001 (5)	−0.001 (5)
C6C	0.094 (12)	0.032 (5)	0.018 (5)	−0.013 (6)	0.020 (5)	0.004 (4)
N7B	0.041 (3)	0.047 (3)	0.115 (5)	−0.003 (2)	−0.009 (3)	0.002 (3)
C8B	0.035 (3)	0.044 (3)	0.089 (5)	0.005 (2)	−0.015 (3)	−0.004 (3)
N9B	0.047 (3)	0.044 (3)	0.053 (3)	−0.004 (2)	0.011 (2)	−0.008 (2)
O3WA	0.054 (12)	0.070 (6)	0.065 (7)	−0.018 (5)	0.002 (9)	0.012 (5)
O3WB	0.039 (13)	0.056 (6)	0.032 (6)	0.001 (6)	−0.005 (5)	−0.005 (5)
S1A	0.0190 (5)	0.0406 (6)	0.0304 (5)	0.0041 (4)	0.0014 (4)	0.0024 (5)
O7A	0.0204 (15)	0.082 (3)	0.0395 (19)	0.0058 (18)	0.0025 (14)	0.0025 (19)
O8A	0.0254 (16)	0.081 (3)	0.040 (2)	0.0042 (17)	−0.0062 (14)	0.0015 (19)
O9A	0.0374 (19)	0.064 (2)	0.0339 (18)	0.0009 (19)	−0.0081 (15)	0.0020 (18)
O10A	0.0233 (15)	0.048 (2)	0.045 (2)	−0.0038 (14)	−0.0008 (14)	0.0051 (16)
O11A	0.0363 (19)	0.045 (2)	0.058 (2)	0.0119 (16)	−0.0007 (17)	0.0030 (18)
O12A	0.0297 (17)	0.066 (2)	0.0331 (18)	0.0037 (17)	0.0043 (14)	0.0005 (17)
C9A	0.024 (2)	0.036 (2)	0.033 (2)	−0.0022 (18)	−0.0001 (17)	0.0048 (19)
C10A	0.0214 (19)	0.029 (2)	0.038 (2)	0.0005 (17)	0.0022 (17)	0.0051 (19)
C11A	0.032 (2)	0.031 (2)	0.033 (2)	0.0002 (19)	−0.0038 (18)	0.0015 (19)
C12A	0.036 (2)	0.048 (3)	0.031 (2)	0.001 (2)	0.0036 (19)	−0.001 (2)
C13A	0.024 (2)	0.044 (3)	0.038 (2)	0.005 (2)	0.0063 (18)	−0.004 (2)
C14A	0.024 (2)	0.031 (2)	0.035 (2)	0.0021 (17)	−0.0004 (17)	−0.0004 (19)
C15A	0.024 (2)	0.032 (2)	0.031 (2)	0.0006 (17)	0.0008 (16)	−0.0007 (18)
S1B	0.0199 (5)	0.0449 (7)	0.0374 (6)	0.0012 (5)	−0.0017 (4)	0.0018 (5)
O7B	0.0216 (15)	0.080 (3)	0.0314 (17)	−0.0009 (17)	−0.0012 (13)	0.0034 (18)
O8B	0.0331 (18)	0.080 (3)	0.0366 (19)	−0.0020 (18)	0.0085 (15)	−0.0028 (19)
O9B	0.048 (2)	0.069 (3)	0.0303 (18)	0.000 (2)	0.0063 (15)	−0.0004 (18)
O10B	0.0261 (17)	0.053 (2)	0.060 (2)	0.0086 (16)	−0.0035 (16)	−0.0015 (19)
O11B	0.041 (2)	0.052 (2)	0.070 (3)	−0.0094 (18)	0.0085 (19)	0.003 (2)
O12B	0.0287 (17)	0.088 (3)	0.0354 (19)	0.0048 (19)	−0.0048 (14)	−0.003 (2)
C9B	0.029 (2)	0.036 (2)	0.030 (2)	0.0049 (19)	0.0029 (18)	0.0043 (19)
C10B	0.025 (2)	0.029 (2)	0.030 (2)	0.0021 (17)	−0.0024 (16)	0.0034 (18)
C11B	0.038 (2)	0.035 (2)	0.027 (2)	0.000 (2)	0.0025 (18)	0.0020 (19)
C12B	0.039 (3)	0.050 (3)	0.032 (2)	−0.002 (2)	−0.007 (2)	−0.006 (2)
C13B	0.027 (2)	0.041 (3)	0.041 (3)	−0.003 (2)	−0.0124 (19)	−0.002 (2)
C14B	0.025 (2)	0.031 (2)	0.030 (2)	−0.0007 (17)	−0.0004 (16)	0.0033 (19)
C15B	0.0243 (19)	0.029 (2)	0.027 (2)	0.0013 (17)	−0.0014 (16)	−0.0012 (18)
O1W	0.0373 (19)	0.064 (2)	0.0336 (18)	−0.0042 (19)	0.0112 (16)	0.0006 (17)
O2W	0.0378 (19)	0.057 (2)	0.0288 (17)	0.0041 (17)	0.0081 (14)	0.0002 (17)
O4W	0.165 (6)	0.065 (3)	0.067 (3)	0.006 (4)	−0.048 (4)	−0.014 (3)

### 6-Oxo-1,9-dihydropurin-7-ium 5-sulfosalicylate dihydrate (I) Geometric parameters (Å, º)

**Table:**

O6A—C6A	1.228 (5)	S1A—O12A	1.453 (3)
N1A—C2A	1.346 (6)	S1A—O10A	1.466 (3)
N1A—C6A	1.395 (5)	S1A—C14A	1.772 (4)
N1A—H1A	0.8600	O7A—C9A	1.318 (6)
N3A—C2A	1.293 (6)	O7A—H7D	0.8200
N3A—C4A	1.362 (5)	O8A—C9A	1.216 (5)
N7A—C8A	1.330 (6)	O9A—C11A	1.350 (5)
N7A—C5A	1.384 (5)	O9A—H9D	0.8200
N7A—H7A	0.8600	C9A—C10A	1.479 (6)
N9A—C8A	1.334 (6)	C10A—C15A	1.389 (6)
N9A—C4A	1.370 (5)	C10A—C11A	1.402 (6)
N9A—H9A	0.8600	C11A—C12A	1.395 (6)
C2A—H2A	0.9300	C12A—C13A	1.378 (7)
C4A—C5A	1.354 (6)	C12A—H12A	0.9300
C5A—C6A	1.415 (6)	C13A—C14A	1.394 (6)
C8A—H8A	0.9300	C13A—H13A	0.9300
N1B—C2B	1.300 (9)	C14A—C15A	1.382 (6)
N1B—C6B	1.452 (12)	C15A—H15A	0.9300
N1B—C5B	1.452 (15)	S1B—O11B	1.447 (4)
N1B—H1C	0.8600	S1B—O12B	1.456 (4)
N1B—H1B	0.8600	S1B—O10B	1.460 (4)
C2B—N3B	1.301 (7)	S1B—C14B	1.767 (4)
C2B—C5B	2.013 (16)	O7B—C9B	1.310 (5)
C2B—H2B	0.9300	O7B—H7E	0.8200
N3B—C4B	1.361 (6)	O8B—C9B	1.227 (5)
C4B—N9B	1.339 (6)	O9B—C11B	1.342 (6)
C4B—C5B	1.378 (12)	O9B—H9E	0.8200
C4B—C5C	1.396 (12)	C9B—C10B	1.466 (6)
O6B—C6B	1.222 (15)	C10B—C15B	1.401 (6)
C5C—N7B	1.425 (13)	C10B—C11B	1.405 (6)
C5C—C6B	1.458 (18)	C11B—C12B	1.399 (7)
C5C—C8B	2.009 (15)	C12B—C13B	1.366 (7)
O6C—C6C	1.227 (16)	C12B—H12B	0.9300
C5B—C6C	1.41 (2)	C13B—C14B	1.401 (6)
C6C—N7B	1.476 (14)	C13B—H13B	0.9300
N7B—C8B	1.319 (9)	C14B—C15B	1.383 (6)
N7B—H7B	0.8600	C15B—H15B	0.9300
N7B—H7C	0.8600	O1W—H1WA	0.8501
C8B—N9B	1.301 (7)	O1W—H1WB	0.8493
C8B—H8B	0.9300	O2W—H2WA	0.821 (10)
N9B—H9B	0.8600	O2W—H2WB	0.820 (10)
S1A—O11A	1.439 (4)		
			
C2A—N1A—C6A	125.2 (4)	N9B—C8B—C5C	74.8 (5)
C2A—N1A—H1A	117.4	N7B—C8B—C5C	45.0 (4)
C6A—N1A—H1A	117.4	N9B—C8B—H8B	120.1
C2A—N3A—C4A	112.2 (4)	N7B—C8B—H8B	120.1
C8A—N7A—C5A	107.1 (4)	C5C—C8B—H8B	165.1
C8A—N7A—H7A	126.4	C8B—N9B—C4B	109.5 (5)
C5A—N7A—H7A	126.4	C8B—N9B—H9B	125.2
C8A—N9A—C4A	107.7 (4)	C4B—N9B—H9B	125.2
C8A—N9A—H9A	126.1	O11A—S1A—O12A	112.6 (2)
C4A—N9A—H9A	126.1	O11A—S1A—O10A	113.0 (2)
N3A—C2A—N1A	125.4 (4)	O12A—S1A—O10A	111.8 (2)
N3A—C2A—H2A	117.3	O11A—S1A—C14A	106.4 (2)
N1A—C2A—H2A	117.3	O12A—S1A—C14A	106.0 (2)
C5A—C4A—N3A	126.3 (4)	O10A—S1A—C14A	106.4 (2)
C5A—C4A—N9A	107.5 (4)	C9A—O7A—H7D	109.5
N3A—C4A—N9A	126.2 (4)	C11A—O9A—H9D	109.5
C4A—C5A—N7A	107.5 (4)	O8A—C9A—O7A	122.1 (4)
C4A—C5A—C6A	121.5 (4)	O8A—C9A—C10A	123.6 (4)
N7A—C5A—C6A	131.0 (4)	O7A—C9A—C10A	114.2 (4)
O6A—C6A—N1A	121.4 (4)	C15A—C10A—C11A	119.5 (4)
O6A—C6A—C5A	129.1 (4)	C15A—C10A—C9A	121.1 (4)
N1A—C6A—C5A	109.5 (4)	C11A—C10A—C9A	119.4 (4)
N7A—C8A—N9A	110.1 (4)	O9A—C11A—C12A	116.8 (4)
N7A—C8A—H8A	125.0	O9A—C11A—C10A	123.8 (4)
N9A—C8A—H8A	125.0	C12A—C11A—C10A	119.4 (4)
C2B—N1B—C6B	137.0 (7)	C13A—C12A—C11A	120.5 (4)
C2B—N1B—C5B	93.9 (7)	C13A—C12A—H12A	119.8
C2B—N1B—H1C	111.5	C11A—C12A—H12A	119.8
C6B—N1B—H1C	111.5	C12A—C13A—C14A	120.2 (4)
C2B—N1B—H1B	133.1	C12A—C13A—H13A	119.9
C5B—N1B—H1B	133.1	C14A—C13A—H13A	119.9
N1B—C2B—N3B	121.5 (6)	C15A—C14A—C13A	119.7 (4)
N1B—C2B—C5B	46.0 (5)	C15A—C14A—S1A	121.3 (3)
N3B—C2B—C5B	75.5 (5)	C13A—C14A—S1A	119.0 (3)
N1B—C2B—H2B	119.3	C14A—C15A—C10A	120.7 (4)
N3B—C2B—H2B	119.3	C14A—C15A—H15A	119.6
C5B—C2B—H2B	165.3	C10A—C15A—H15A	119.6
C2B—N3B—C4B	107.9 (5)	O11B—S1B—O12B	112.8 (3)
N9B—C4B—N3B	126.2 (5)	O11B—S1B—O10B	112.5 (2)
N9B—C4B—C5B	133.4 (8)	O12B—S1B—O10B	111.5 (2)
N3B—C4B—C5B	100.4 (7)	O11B—S1B—C14B	106.2 (2)
N9B—C4B—C5C	99.5 (7)	O12B—S1B—C14B	107.3 (2)
N3B—C4B—C5C	134.3 (7)	O10B—S1B—C14B	106.1 (2)
C4B—C5C—N7B	117.1 (10)	C9B—O7B—H7E	109.5
C4B—C5C—C6B	117.7 (10)	C11B—O9B—H9E	109.5
N7B—C5C—C6B	125.2 (10)	O8B—C9B—O7B	122.7 (4)
C4B—C5C—C8B	76.2 (6)	O8B—C9B—C10B	122.9 (4)
N7B—C5C—C8B	40.9 (5)	O7B—C9B—C10B	114.4 (4)
C6B—C5C—C8B	166.0 (9)	C15B—C10B—C11B	119.4 (4)
O6B—C6B—N1B	136.7 (11)	C15B—C10B—C9B	121.3 (4)
O6B—C6B—C5C	121.8 (11)	C11B—C10B—C9B	119.3 (4)
N1B—C6B—C5C	101.5 (8)	O9B—C11B—C12B	117.6 (4)
C4B—C5B—C6C	120.4 (12)	O9B—C11B—C10B	122.8 (4)
C4B—C5B—N1B	116.4 (10)	C12B—C11B—C10B	119.6 (4)
C6C—C5B—N1B	123.2 (10)	C13B—C12B—C11B	120.5 (4)
C4B—C5B—C2B	76.3 (7)	C13B—C12B—H12B	119.8
C6C—C5B—C2B	163.3 (10)	C11B—C12B—H12B	119.8
N1B—C5B—C2B	40.1 (5)	C12B—C13B—C14B	120.5 (4)
O6C—C6C—C5B	126.6 (13)	C12B—C13B—H13B	119.8
O6C—C6C—N7B	132.2 (13)	C14B—C13B—H13B	119.8
C5B—C6C—N7B	101.2 (8)	C15B—C14B—C13B	119.8 (4)
C8B—N7B—C5C	94.1 (7)	C15B—C14B—S1B	120.8 (3)
C8B—N7B—C6C	135.6 (7)	C13B—C14B—S1B	119.4 (3)
C8B—N7B—H7B	133.0	C14B—C15B—C10B	120.2 (4)
C5C—N7B—H7B	133.0	C14B—C15B—H15B	119.9
C8B—N7B—H7C	112.2	C10B—C15B—H15B	119.9
C6C—N7B—H7C	112.2	H1WA—O1W—H1WB	104.5
N9B—C8B—N7B	119.8 (6)	H2WA—O2W—H2WB	112 (10)
			
C4A—N3A—C2A—N1A	0.3 (7)	C6B—C5C—N7B—C8B	178.1 (9)
C6A—N1A—C2A—N3A	−0.5 (8)	O6C—C6C—N7B—C8B	178.6 (13)
C2A—N3A—C4A—C5A	0.0 (7)	C5B—C6C—N7B—C8B	−2.0 (12)
C2A—N3A—C4A—N9A	−179.1 (4)	C5C—N7B—C8B—N9B	0.3 (8)
C8A—N9A—C4A—C5A	−0.4 (5)	C6C—N7B—C8B—N9B	2.6 (12)
C8A—N9A—C4A—N3A	179.0 (4)	N7B—C8B—N9B—C4B	−0.6 (8)
N3A—C4A—C5A—N7A	−179.2 (4)	C5C—C8B—N9B—C4B	−0.4 (5)
N9A—C4A—C5A—N7A	0.1 (5)	N3B—C4B—N9B—C8B	178.7 (5)
N3A—C4A—C5A—C6A	−0.1 (7)	C5B—C4B—N9B—C8B	−1.3 (10)
N9A—C4A—C5A—C6A	179.2 (4)	C5C—C4B—N9B—C8B	0.5 (7)
C8A—N7A—C5A—C4A	0.2 (5)	O8A—C9A—C10A—C15A	179.1 (5)
C8A—N7A—C5A—C6A	−178.8 (5)	O7A—C9A—C10A—C15A	1.1 (6)
C2A—N1A—C6A—O6A	−179.1 (5)	O8A—C9A—C10A—C11A	−0.3 (7)
C2A—N1A—C6A—C5A	0.4 (6)	O7A—C9A—C10A—C11A	−178.2 (4)
C4A—C5A—C6A—O6A	179.4 (5)	C15A—C10A—C11A—O9A	−180.0 (4)
N7A—C5A—C6A—O6A	−1.7 (9)	C9A—C10A—C11A—O9A	−0.6 (7)
C4A—C5A—C6A—N1A	−0.1 (6)	C15A—C10A—C11A—C12A	−1.3 (7)
N7A—C5A—C6A—N1A	178.7 (4)	C9A—C10A—C11A—C12A	178.1 (4)
C5A—N7A—C8A—N9A	−0.4 (6)	O9A—C11A—C12A—C13A	179.2 (5)
C4A—N9A—C8A—N7A	0.5 (6)	C10A—C11A—C12A—C13A	0.4 (7)
C6B—N1B—C2B—N3B	0.6 (12)	C11A—C12A—C13A—C14A	0.6 (8)
C5B—N1B—C2B—N3B	−0.6 (8)	C12A—C13A—C14A—C15A	−0.8 (7)
N1B—C2B—N3B—C4B	0.6 (8)	C12A—C13A—C14A—S1A	178.7 (4)
C5B—C2B—N3B—C4B	0.2 (5)	O11A—S1A—C14A—C15A	116.8 (4)
C2B—N3B—C4B—N9B	179.7 (5)	O12A—S1A—C14A—C15A	−3.4 (5)
C2B—N3B—C4B—C5B	−0.2 (7)	O10A—S1A—C14A—C15A	−122.5 (4)
C2B—N3B—C4B—C5C	−2.7 (10)	O11A—S1A—C14A—C13A	−62.7 (4)
N9B—C4B—C5C—N7B	−0.4 (9)	O12A—S1A—C14A—C13A	177.2 (4)
N3B—C4B—C5C—N7B	−178.3 (6)	O10A—S1A—C14A—C13A	58.0 (4)
N9B—C4B—C5C—C6B	−178.6 (8)	C13A—C14A—C15A—C10A	−0.1 (7)
N3B—C4B—C5C—C6B	3.4 (13)	S1A—C14A—C15A—C10A	−179.6 (3)
N9B—C4B—C5C—C8B	−0.3 (4)	C11A—C10A—C15A—C14A	1.1 (7)
N3B—C4B—C5C—C8B	−178.3 (6)	C9A—C10A—C15A—C14A	−178.2 (4)
C2B—N1B—C6B—O6B	−177.8 (13)	O8B—C9B—C10B—C15B	179.5 (5)
C2B—N1B—C6B—C5C	−0.1 (12)	O7B—C9B—C10B—C15B	−1.8 (6)
C4B—C5C—C6B—O6B	176.7 (11)	O8B—C9B—C10B—C11B	−2.8 (7)
N7B—C5C—C6B—O6B	−1.4 (17)	O7B—C9B—C10B—C11B	175.9 (4)
C8B—C5C—C6B—O6B	4 (4)	C15B—C10B—C11B—O9B	179.4 (4)
C4B—C5C—C6B—N1B	−1.5 (11)	C9B—C10B—C11B—O9B	1.6 (7)
N7B—C5C—C6B—N1B	−179.6 (8)	C15B—C10B—C11B—C12B	0.0 (7)
C8B—C5C—C6B—N1B	−174 (3)	C9B—C10B—C11B—C12B	−177.8 (5)
N9B—C4B—C5B—C6C	1.7 (14)	O9B—C11B—C12B—C13B	−178.9 (5)
N3B—C4B—C5B—C6C	−178.4 (9)	C10B—C11B—C12B—C13B	0.5 (8)
N9B—C4B—C5B—N1B	179.9 (6)	C11B—C12B—C13B—C14B	−1.1 (8)
N3B—C4B—C5B—N1B	−0.1 (9)	C12B—C13B—C14B—C15B	1.2 (7)
N9B—C4B—C5B—C2B	−179.8 (6)	C12B—C13B—C14B—S1B	−177.9 (4)
N3B—C4B—C5B—C2B	0.1 (4)	O11B—S1B—C14B—C15B	−114.7 (4)
C2B—N1B—C5B—C4B	0.4 (9)	O12B—S1B—C14B—C15B	6.0 (5)
C2B—N1B—C5B—C6C	178.6 (9)	O10B—S1B—C14B—C15B	125.3 (4)
C4B—C5B—C6C—O6C	179.4 (12)	O11B—S1B—C14B—C13B	64.3 (4)
N1B—C5B—C6C—O6C	1.2 (19)	O12B—S1B—C14B—C13B	−174.9 (4)
C2B—C5B—C6C—O6C	4 (4)	O10B—S1B—C14B—C13B	−55.6 (4)
C4B—C5B—C6C—N7B	0.0 (12)	C13B—C14B—C15B—C10B	−0.6 (7)
N1B—C5B—C6C—N7B	−178.2 (8)	S1B—C14B—C15B—C10B	178.4 (3)
C2B—C5B—C6C—N7B	−175 (3)	C11B—C10B—C15B—C14B	0.0 (7)
C4B—C5C—N7B—C8B	0.1 (9)	C9B—C10B—C15B—C14B	177.8 (4)

### 6-Oxo-1,9-dihydropurin-7-ium 5-sulfosalicylate dihydrate (I) Hydrogen-bond geometry (Å, º)

**Table:**

*D*—H···*A*	*D*—H	H···*A*	*D*···*A*	*D*—H···*A*
N7*B*—H7*B*···O3*WA*^i^	0.86	2.26	3.08	158
O7*A*—H7*D*···O10*A*^ii^	0.82	1.86	2.677	170
O7*B*—H7*E*···O10*B*^i^	0.82	1.84	2.655	175
O9*A*—H9*D*···O12*B*^ii^	0.82	2.34	2.924	128
O9*B*—H9*E*···O12*A*^iii^	0.82	2.54	3.143	131
O1*W*—H1*WA*···O6*A*^iv^	0.85	2.31	2.801	117
O1*W*—H1*WA*···O10*B*^iv^	0.85	2.28	2.917	132
N9*B*—H9*B*···O6*B*^v^	0.86	2.42	3.044	130
N9*B*—H9*B*···O3*WA*^vi^	0.86	2.47	3.07	128
N1*A*—H1*A*···O6*A*^vii^	0.86	2.05	2.898	170
N1*B*—H1*C*···O4*W*	0.86	2.22	2.890	135
N1*B*—H1*C*···O11*A*	0.86	2.45	2.998	122
O1*W*—H1*WB*···O12*B*	0.85	2.01	2.844	169
O2*W*—H2*WA*···N3*A*	0.83	2.07	2.849	157
O2*W*—H2*WB*···O12*A*	0.82	2.03	2.815	160
N7*A*—H7*A*···O1*W*	0.86	1.77	2.615	168
N9*A*—H9*A*···O2*W*	0.86	1.89	2.697	157
C2*A*—H2*A*···O1*W*^ii^	0.93	2.43	3.149	134
C2*B*—H2*B*···O11*A*	0.93	2.46	2.974	114
C8*A*—H8*A*···O2*W*^viii^	0.93	2.40	3.310	167
C15*B*—H15*B*···O9*A*	0.93	2.59	3.510	172

Symmetry codes: (i) *x*+1, *y*, *z*; (ii) *x*−1, *y*, *z*; (iii) *x*+1, *y*, *z*−1; (iv) −*x*+1, −*y*+1, −*z*+1; (v) *x*, −*y*+1/2, *z*−1/2; (vi) *x*+1, −*y*+1/2, *z*−1/2; (vii) −*x*, −*y*+1, −*z*+1; (viii) −*x*+1, −*y*+1, −*z*+2.

### 6-Oxo-1,9-dihydropurin-7-ium perchlorate monohydrate (II) Crystal data

**Table:**

C_5_H_5_N_4_O^+^·ClO_4_^−^·H_2_O	*F*(000) = 520
*M**_r_* = 254.60	*D*_x_ = 1.833 Mg m^−^^3^
Monoclinic, *P*2_1_/*c*	Mo *K*α radiation, λ = 0.71073 Å
*a* = 5.0307 (6) Å	Cell parameters from 2752 reflections
*b* = 20.386 (2) Å	θ = 2.0–30.3°
*c* = 9.0181 (10) Å	µ = 0.44 mm^−^^1^
β = 94.233 (2)°	*T* = 296 K
*V* = 922.33 (18) Å^3^	Plate, colourless
*Z* = 4	0.45 × 0.02 × 0.003 mm

### 6-Oxo-1,9-dihydropurin-7-ium perchlorate monohydrate (II) Data collection

**Table:**

Bruker APEXII CCD diffractometer	2370 reflections with *I* > 2σ(*I*)
φ and ω scans	*R*_int_ = 0.025
Absorption correction: multi-scan (*SADABS*; Bruker, 2016)	θ_max_ = 30.3°, θ_min_ = 2.0°
*T*_min_ = 0.957, *T*_max_ = 1.000	*h* = −7→7
16360 measured reflections	*k* = −28→28
2752 independent reflections	*l* = −12→12

### 6-Oxo-1,9-dihydropurin-7-ium perchlorate monohydrate (II) Refinement

**Table:**

Refinement on *F*^2^	3 restraints
Least-squares matrix: full	Hydrogen site location: mixed
*R*[*F*^2^ > 2σ(*F*^2^)] = 0.038	H atoms treated by a mixture of independent and constrained refinement
*wR*(*F*^2^) = 0.111	*w* = 1/[σ^2^(*F*_o_^2^) + (0.0576*P*)^2^ + 0.3728*P*] where *P* = (*F*_o_^2^ + 2*F*_c_^2^)/3
*S* = 1.05	(Δ/σ)_max_ < 0.001
2752 reflections	Δρ_max_ = 0.37 e Å^−^^3^
165 parameters	Δρ_min_ = −0.29 e Å^−^^3^

### 6-Oxo-1,9-dihydropurin-7-ium perchlorate monohydrate (II) Special details

**Table:**

Geometry. All esds (except the esd in the dihedral angle between two l.s. planes) are estimated using the full covariance matrix. The cell esds are taken into account individually in the estimation of esds in distances, angles and torsion angles; correlations between esds in cell parameters are only used when they are defined by crystal symmetry. An approximate (isotropic) treatment of cell esds is used for estimating esds involving l.s. planes.

### 6-Oxo-1,9-dihydropurin-7-ium perchlorate monohydrate (II) Fractional atomic coordinates and isotropic or equivalent isotropic displacement parameters (Å^2^)

**Table:**

	*x*	*y*	*z*	*U*_iso_*/*U*_eq_	
Cl1	1.32826 (7)	0.47612 (2)	0.23036 (5)	0.03437 (13)	
O6	1.1975 (2)	0.28099 (6)	0.28926 (13)	0.0395 (3)	
O2	1.2250 (4)	0.53538 (8)	0.1690 (2)	0.0699 (5)	
O3	1.6141 (3)	0.47706 (8)	0.2342 (2)	0.0627 (4)	
O4	1.2288 (3)	0.42071 (7)	0.14577 (18)	0.0571 (4)	
O5	1.2456 (3)	0.46991 (8)	0.37946 (17)	0.0598 (4)	
N1	0.9409 (3)	0.35230 (6)	0.41720 (15)	0.0334 (3)	
H1	1.018 (4)	0.3843 (12)	0.387 (3)	0.049 (6)*	
N7	0.8829 (2)	0.17434 (6)	0.43852 (14)	0.0281 (3)	
H7	0.992 (5)	0.1492 (12)	0.387 (3)	0.060 (7)*	
N9	0.5789 (2)	0.20049 (6)	0.58838 (14)	0.0284 (3)	
H9	0.462 (5)	0.1993 (11)	0.650 (3)	0.046 (6)*	
C2	0.7509 (3)	0.36488 (7)	0.51258 (18)	0.0342 (3)	
H2	0.716229	0.408591	0.533422	0.041*	
N3	0.6127 (3)	0.32029 (6)	0.57756 (14)	0.0312 (3)	
C4	0.6819 (3)	0.25866 (7)	0.53983 (15)	0.0246 (3)	
C5	0.8730 (3)	0.24168 (7)	0.44524 (15)	0.0243 (3)	
C6	1.0223 (3)	0.29051 (7)	0.37512 (16)	0.0272 (3)	
C8	0.7053 (3)	0.15077 (7)	0.52526 (17)	0.0311 (3)	
H8	0.672604	0.106462	0.540420	0.037*	
O1W	0.2214 (3)	0.38054 (6)	0.76425 (18)	0.0511 (4)	
H1W	0.252 (5)	0.4216 (5)	0.765 (3)	0.066 (7)*	
H2W	0.344 (4)	0.3627 (10)	0.718 (3)	0.081 (9)*	

### 6-Oxo-1,9-dihydropurin-7-ium perchlorate monohydrate (II) Atomic displacement parameters (Å^2^)

**Table:**

	*U*^11^	*U*^22^	*U*^33^	*U*^12^	*U*^13^	*U*^23^
Cl1	0.0347 (2)	0.02074 (17)	0.0495 (2)	−0.00172 (12)	0.01561 (16)	−0.00075 (13)
O6	0.0398 (6)	0.0391 (6)	0.0428 (6)	−0.0012 (5)	0.0248 (5)	0.0054 (5)
O2	0.0807 (12)	0.0369 (7)	0.0952 (13)	0.0172 (7)	0.0277 (10)	0.0222 (8)
O3	0.0351 (7)	0.0545 (9)	0.1004 (13)	−0.0050 (6)	0.0182 (7)	−0.0109 (8)
O4	0.0551 (8)	0.0419 (8)	0.0757 (10)	−0.0102 (6)	0.0147 (7)	−0.0226 (7)
O5	0.0734 (10)	0.0587 (9)	0.0503 (8)	−0.0211 (8)	0.0244 (7)	−0.0040 (6)
N1	0.0377 (7)	0.0255 (6)	0.0388 (7)	−0.0043 (5)	0.0146 (5)	0.0030 (5)
N7	0.0305 (6)	0.0236 (5)	0.0317 (6)	0.0031 (4)	0.0130 (5)	0.0006 (4)
N9	0.0282 (6)	0.0285 (6)	0.0304 (6)	−0.0020 (4)	0.0141 (5)	0.0016 (4)
C2	0.0410 (8)	0.0242 (6)	0.0385 (8)	0.0007 (6)	0.0120 (6)	−0.0031 (6)
N3	0.0331 (6)	0.0269 (6)	0.0351 (6)	0.0024 (5)	0.0132 (5)	−0.0026 (5)
C4	0.0233 (6)	0.0261 (6)	0.0254 (6)	−0.0003 (5)	0.0075 (5)	0.0005 (5)
C5	0.0246 (6)	0.0241 (6)	0.0251 (6)	0.0007 (5)	0.0085 (5)	0.0023 (5)
C6	0.0269 (6)	0.0279 (6)	0.0277 (6)	−0.0020 (5)	0.0083 (5)	0.0032 (5)
C8	0.0338 (7)	0.0245 (6)	0.0367 (7)	−0.0007 (5)	0.0128 (6)	0.0022 (5)
O1W	0.0546 (8)	0.0302 (6)	0.0739 (9)	−0.0081 (6)	0.0417 (7)	−0.0050 (6)

### 6-Oxo-1,9-dihydropurin-7-ium perchlorate monohydrate (II) Geometric parameters (Å, º)

**Table:**

Cl1—O2	1.4116 (15)	N9—C8	1.3452 (19)
Cl1—O4	1.4324 (13)	N9—C4	1.3784 (17)
Cl1—O3	1.4359 (15)	N9—H9	0.84 (2)
Cl1—O5	1.4421 (15)	C2—N3	1.309 (2)
O6—C6	1.2307 (17)	C2—H2	0.9300
N1—C2	1.357 (2)	N3—C4	1.3539 (18)
N1—C6	1.3860 (19)	C4—C5	1.3758 (17)
N1—H1	0.82 (2)	C5—C6	1.4229 (18)
N7—C8	1.3204 (18)	C8—H8	0.9300
N7—C5	1.3752 (18)	O1W—H1W	0.852 (9)
N7—H7	0.90 (3)	O1W—H2W	0.850 (9)
			
O2—Cl1—O4	111.24 (12)	N3—C2—H2	117.4
O2—Cl1—O3	109.68 (11)	N1—C2—H2	117.4
O4—Cl1—O3	109.46 (9)	C2—N3—C4	112.14 (12)
O2—Cl1—O5	108.50 (11)	N3—C4—C5	126.43 (12)
O4—Cl1—O5	108.29 (9)	N3—C4—N9	127.50 (12)
O3—Cl1—O5	109.63 (11)	C5—C4—N9	106.07 (12)
C2—N1—C6	125.56 (13)	N7—C5—C4	107.91 (11)
C2—N1—H1	115.8 (16)	N7—C5—C6	131.06 (12)
C6—N1—H1	118.5 (16)	C4—C5—C6	121.02 (13)
C8—N7—C5	108.00 (12)	O6—C6—N1	123.73 (13)
C8—N7—H7	124.0 (16)	O6—C6—C5	126.53 (14)
C5—N7—H7	128.0 (16)	N1—C6—C5	109.75 (12)
C8—N9—C4	108.26 (11)	N7—C8—N9	109.76 (12)
C8—N9—H9	129.5 (15)	N7—C8—H8	125.1
C4—N9—H9	122.2 (15)	N9—C8—H8	125.1
N3—C2—N1	125.11 (14)	H1W—O1W—H2W	106.9 (14)
			
C6—N1—C2—N3	−1.1 (3)	N3—C4—C5—C6	0.0 (2)
N1—C2—N3—C4	0.5 (2)	N9—C4—C5—C6	−179.54 (13)
C2—N3—C4—C5	0.0 (2)	C2—N1—C6—O6	−179.27 (16)
C2—N3—C4—N9	179.42 (15)	C2—N1—C6—C5	1.0 (2)
C8—N9—C4—N3	−179.25 (14)	N7—C5—C6—O6	0.6 (3)
C8—N9—C4—C5	0.24 (16)	C4—C5—C6—O6	179.83 (15)
C8—N7—C5—C4	0.01 (17)	N7—C5—C6—N1	−179.65 (14)
C8—N7—C5—C6	179.31 (15)	C4—C5—C6—N1	−0.4 (2)
N3—C4—C5—N7	179.34 (14)	C5—N7—C8—N9	0.15 (18)
N9—C4—C5—N7	−0.15 (15)	C4—N9—C8—N7	−0.24 (18)

### 6-Oxo-1,9-dihydropurin-7-ium perchlorate monohydrate (II) Hydrogen-bond geometry (Å, º)

**Table:**

*D*—H···*A*	*D*—H	H···*A*	*D*···*A*	*D*—H···*A*
N1—H1···O4	0.82	2.60	3.249	138
N1—H1···O5	0.82	2.09	2.879	162
N7—H7···O2^i^	0.91	2.60	3.031	110.2
N7—H7···O1*W*^ii^	0.91	1.76	2.6489	165
N9—H9···O6^iii^	0.84	1.93	2.7602	166
O1*W*—H1*W*···O3^iv^	0.85	2.17	3.018	172
O1*W*—H2*W*···N3	0.85	2.11	2.951	172
C8—H8···O2^i^	0.93	2.47	2.970	114
C8—H8···O3^iii^	0.93	2.47	3.268	144
C8—H8···O4^iii^	0.93	2.55	3.072	116

Symmetry codes: (i) −*x*+2, *y*−1/2, −*z*+1/2; (ii) *x*+1, −*y*+1/2, *z*−1/2; (iii) *x*−1, −*y*+1/2, *z*+1/2; (iv) −*x*+2, −*y*+1, −*z*+1.
